# Atrophy Resistant vs. Atrophy Susceptible Skeletal Muscles: “aRaS” as a Novel Experimental Paradigm to Study the Mechanisms of Human Disuse Atrophy

**DOI:** 10.3389/fphys.2021.653060

**Published:** 2021-05-04

**Authors:** Joseph J. Bass, Edward J. O. Hardy, Thomas B. Inns, Daniel J. Wilkinson, Mathew Piasecki, Robert H. Morris, Abi Spicer, Craig Sale, Ken Smith, Philip J. Atherton, Bethan E. Phillips

**Affiliations:** ^1^MRC-Versus Arthritis Centre for Musculoskeletal Ageing Research and National Institute for Health Research (NIHR), Nottingham Biomedical Research Centre (BRC), University of Nottingham, Nottingham, United Kingdom; ^2^Department of Surgery and Anaesthetics, Royal Derby Hospital, Derby, United Kingdom; ^3^School of Science and Technology, Nottingham Trent University, Nottingham, United Kingdom; ^4^Musculoskeletal Physiology Research Group, Sport, Health and Performance Enhancement Research Centre, Nottingham Trent University, Nottingham, United Kingdom

**Keywords:** skeletal muscle atrophy, aRaS, disuse atrophy, tibialis anterior muscle, medial gastrocnemius muscle

## Abstract

**Objective:**

Disuse atrophy (DA) describes inactivity-induced skeletal muscle loss, through incompletely defined mechanisms. An intriguing observation is that individual muscles exhibit differing degrees of atrophy, despite exhibiting similar anatomical function/locations. We aimed to develop an innovative experimental paradigm to investigate Atrophy Resistant *tibialis anterior* (TA) and Atrophy Susceptible *medial gastrocnemius* (MG) muscles (aRaS) with a future view of uncovering central mechanisms.

**Method:**

Seven healthy young men (22 ± 1 year) underwent 15 days unilateral leg immobilisation (ULI). Participants had a single leg immobilised using a knee brace and air-boot to fix the leg (75° knee flexion) and ankle in place. Dual-energy X-ray absorptiometry (DXA), MRI and ultrasound scans of the lower leg were taken before and after the immobilisation period to determine changes in muscle mass. Techniques were developed for conchotome and microneedle TA/MG muscle biopsies following immobilisation (both limbs), and preliminary fibre typing analyses was conducted.

**Results:**

TA/MG muscles displayed comparable fibre type distribution of predominantly type I fibres (TA 67 ± 7%, MG 63 ± 5%). Following 15 days immobilisation, MG muscle volume (–2.8 ± 1.4%, *p* < 0.05) and muscle thickness decreased (−12.9 ± 1.6%, *p* < 0.01), with a positive correlation between changes in muscle volume and thickness (R^2^ = 0.31, *p* = 0.038). Importantly, both TA muscle volume and thickness remained unchanged.

**Conclusion:**

The use of this unique “aRaS” paradigm provides an effective and convenient means by which to study the mechanistic basis of divergent DA susceptibility in humans, which may facilitate new mechanistic insights, and by extension, mitigation of skeletal muscle atrophy during human DA.

## Introduction

Skeletal muscle disuse atrophy (DA), manifesting as a loss of muscle mass, occurs for clinically important reasons such as sedentary behaviour, hospitalisation, enforced bed-rest or limb immobilisation for the purposes of recovery after illness or injury. Any prolonged period (>3–4 days) of disuse is linked with DA and an associated decline in muscle strength, metabolic dysfunction and disability (Dirks et al., 2016). Moreover, repeated bouts of DA are associated with progressive punctuated decreases in physical function and development of sarcopenia (Oikawa et al., 2019). The average stay in a United Kingdom acute care hospital is 6 days (WHO, World Health Organization, 2019), with low mobility during this time a considerable health concern, particularly in older adults who have been reported to remain in bed for 83% of their stay (Harness, 1990). Thus, strategies to further understand and counter DA is a major global scientific endeavour.

Bed-rest, step-count reduction, limb-immobilisation (e.g., casting), unilateral lower-limb suspension (ULLS), and even spaceflight are all DA experimental models used in humans (Rudrappa et al., 2016). Spaceflight induced DA occurs rapidly (Akima et al., 2000), with ∼15% muscle loss in knee extensor/flexor and plantar flexor muscles after 2 weeks exposure to microgravity, with similar losses being shown in quadriceps and gastrocnemius muscles (LeBlanc et al., 2000). Head-down bed rest models of unloading have demonstrated similar degrees of muscle loss (Trappe S. et al., 2004; Rittweger et al., 2005), with ∼17% loss of quadriceps volume after 84 days (Trappe S. et al., 2004). However, bed rest studies necessitate tremendous resources including long-term study facilities, manpower and financial costs. Unilateral limb immobilisation (ULI) either by casting or suspension (ULLS) (de Boer et al., 2007; Wall et al., 2014), also induce considerable muscle loss, with quadriceps CSA decreasing by 3.5% in healthy young men after just 5 days of ULI by casting (Dirks et al., 2014). Furthermore, after 14 days ULI, muscle loss has been shown to progress to an ∼8% decline in both CSA (Wall et al., 2014) and volume (Dirks et al., 2014). Notably, ULI provides advantages over bed-rest models, whereby the non-immobilised weight-bearing limb provides an internal control, and does not induce systemic deconditioning which may introduce confounders such as cardiovascular decline (Krasnoff and Painter, 1999).

Studies investigating DA have primarily focused upon the quadriceps muscles; specifically the v*astus lateralis* (VL), perhaps due to historical recognition of its susceptibility to atrophy, superficial location and ease of biopsy sampling (Shanely et al., 2014). However, beyond gross atrophy of whole muscle groups, it is of significant note that susceptibility to DA differs markedly *between* individual muscles, despite e.g., similar anatomical location and functions (albeit with muscles involved in posture generally exhibiting greater degrees of loss; Belavý et al., 2009). For example, recent studies have demonstrated that with 7 days ULI, the VL muscle atrophied to a greater extent than other quadricep muscles, such as the *M. gracilis* (7.2% vs. 2.3%) (Kilroe et al., 2020). This was further exemplified during longer-term (56 day) bed-rest, whereby individual leg muscles display differing rates of DA, independent of muscle size and location (Belavý et al., 2009). Notably, to us, the plantar flexor *medial gastrocnemius* (MG) appeared across prior studies uniformly highly susceptible to DA, with muscle volume loss of > 20% in response to 56 day bed rest. In contrast, we noted that the ankle flexor *tibialis anterior* (TA) only decreased by ∼5% (Belavý et al., 2009; Miokovic et al., 2012). If internally validated, this provides an experimental paradigm vis-à-vis; the assessment of “atrophy resistant” alongside “atrophy susceptible” muscles, which we propose may facilitate elucidation of the most dominant atrophy-driving mechanisms. Hence, we developed a ULI paradigm that can be undertaken to study the mechanistic basis of DA, through simultaneous investigation of an atrophy susceptible (MG) vs. atrophy resistant (TA) muscle (aRaS), which may highlight core regulatory mechanisms to oppose DA.

## Methods

### Participants

Seven healthy young men (22 ± 1 year, BMI 23 ± 1 kg.m^2^) volunteered to participate in this study. Participants were screened by medical questionnaire, physical assessment and resting electrocardiogram, with exclusions for cardiovascular, metabolic (e.g., type 2 diabetes) and respiratory disorders, along with any other symptoms of ill-health. Participants had clinically normal blood chemistry and pressure (<140/90), were not currently on any prescribed medication, undertook typical activities of daily living and recreation, but had not participated in any exercise training regime within the previous 12 months. Further exclusion criteria included recent steroid treatment (within the last 6 months), BMI < 18 or > 35 kg/m^2^, clotting dysfunction and musculoskeletal disorders. All participants provided written, informed consent to participate in the study after all procedures and risks were explained. The study was approved by The University of Nottingham Faculty of Medicine and Health Sciences Research Ethics Committee and conducted according to the declaration of Helsinki. The study was registered online at clinicaltrials.gov (NCT04199923).

### Experimental Procedures

**FIGURE 1:**
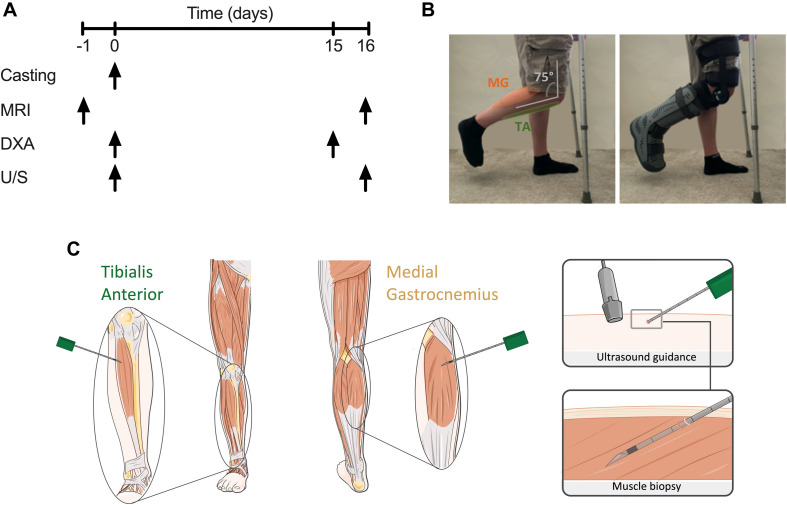
**(A)** Schematic diagram of study protocol; **(B)** unilateral lower leg immobilisation using knee brace and foot boot; **(C)** representative image of microneedle muscle biopsies of *tibialis anterior* (TA) and *medial gastrocnemius* (MG) under ultrasound guidance.

A graphical representation of the experimental design is illustrated in Figure 1A. Following successful screening, participants attended the laboratory for four visits over 16 days, with the period of unilateral leg immobilisation (ULI) spanning 15 days. One day prior to ULI, participants received a whole leg MRI scan on both legs according to the protocol detailed below. On the Day 0 “casting visit,” participants arrived to the laboratory ∼0800 h, following an overnight fast (water *ad libitum*), for a whole-body dual-energy X-ray absorptiometry (DXA) scan and ultrasound (U/S) scan to assess muscle architecture before ULI (Figure 1B). Participants returned to the laboratory (via taxi) for a mid-point visit 5–6 days after ULI to check boot/brace fitting. After 15 days ULI participants returned to the laboratory (via taxi) for a repeat DXA scan, with follow-up MRI and U/S scans taking place the following day. ULI was maintained throughout this 15 day period.

Microneedle and conchotome TA and MG muscle biopsies were taken under sterile conditions from both limbs as proof-of-concept to integrate molecular, metabolic and immunohistochemical analysis (Hayot et al., 2005). Ultrasonography of the MG and TA muscle belly was performed in both transverse and longitudinal planes to identify an area clear of any intra-muscular or superficial veins. The region most suitable for biopsy in MG was located within 10 cm of the knee crease in the medial aspect of the muscle, and in TA was 5–10 cm below the joint line of the knee and 1–2 cm lateral to the border of the tibia. After marking the skin overlying the area identified to be optimal, the skin was shaved, cleaned using Betadine surgical scrub, a sterile field applied to the area and the following steps performed under strict aseptic technique. One milliliter of 1% Lidocaine was infiltrated superficially into the skin at the site identified for biopsy using a 31G needle. Under U/S guidance, 3 ml of 0.5% lidocaine (1.5 ml of 1% lidocaine mixed with 1.5 ml of normal saline) was injected around the upper muscle sheath and intra-muscularly using a 21G needle. Using a No 11 blade a < 5 mm incision was then made in the skin. For microneedle biopsies (Figure 1C), a 10 cm, 12G biopsy needle was mounted into the BARD MAGNUM biopsy device, with the fire depth set to 12 mm. The biopsy needle was inserted through the incision, in a longitudinal direction along the muscle and at a 20–30 degree angle to the skin. After adjusting the needle so that it was identified on the U/S image as within the selected area for biopsy, the needle was inserted through the muscle fascia. With the tip of the needle just below the fascia the biopsy device was fired, in line with the muscle fibres and with the whole length of the needle visible by U/S throughout. The needle was then immediately withdrawn and manual pressure applied to the area. A maximum of 6 needle passes were performed with a minimum of 30 s manual pressure applied between each needle pass giving a total yield of ∼20–50 mg muscle tissue, snap frozen in liquid nitrogen. After the final needle pass manual pressure was applied to the area for 3–5 min. The skin incision was then closed using a steristrip and a gentle pressure dressing applied. Additional conchotome muscle biopsies were undertaken using previously established and standardised techniques (Dietrichson et al., 1987), to collect muscle biopsies of sufficient size for histology analysis. Any fat or connective tissues was dissected from these muscle biopsy samples, and either snap frozen in liquid nitrogen or mounted in optimal cutting temperature (OCT) compound and frozen in liquid nitrogen-cooled isopentane, before storage at −80°C.

### Unilateral Leg Immobilisation

Unilateral leg immobilisation was achieved through use of a hinged leg brace (Knee Post op Cool, Össur, Iceland) and air-boot (Rebound Air Walker, Össur), with the participant ambulating on crutches throughout the immobilisation period (after sufficient training) and asked to continue weight-bearing on the non-immobilised leg. The participants dominant leg (e.g., which would be used to kick a ball) was immobilised, with the remaining non-immobilised leg acting as a within-participant control. The hinged leg brace was fitted over a compression sock with the supplied foam straps, around the thigh and lower leg and fixed at 75° knee flexion (see Figure 1B) to ensure no weight bearing could occur and allow sufficient ground clearance of the air-boot. The participant’s immobilised leg was subsequently placed into the air-boot, with the heel placed at the back of the boot, before the foot was securely fixed into place. This resulted in a fixed neutral ankle position, ensuring no plantar flexion and lengthening of the TA occurred, with combined fixing of the knee at 75° flexion stopping MG lengthening. Ankle motion was checked, with straps adjusted and boot lining inflated to ensure minimal horizonal and vertical movement. “Tamper tags” with the investigators signature were placed on the bootstraps, to indicate if the boot or brace had been modified. Breaking of the tags would result in the participant being excluded. Participants were instructed to ensure no weight bearing occurred on the immobilised leg, to refrain from ankle movement. Where possible participants were asked to continue with daily activities (e.g., traveling to work, cooking), but undertake no formal exercise. Participants were provided with waterproof covers to wear over the brace and boot when showering. Daily contact was maintained with the participants, with brace and boot fitting and compliance checked mid-way through the investigation during a mid-point visit. All participants complied with immobilisation instruction and none were excluded, and returned to normal daily activities after immobilisation.

### Muscle Histology

Serial 7μm thick TA and MG cross-sections from 4 participants were cut at −22°C using a cryostat (Leica, Germany) and mounted on glass slides before air-drying. Determination of muscle fibre types was performed using modified ATPase histochemical staining (Brooke and Kaiser, 1970). Briefly, muscle sections were incubated in buffer containing 100 mM glycine, 100 mM NaCl, 125 mM CaCl_2_ and 1M ATP (pH 9.7) at 37°C for 15 min, before being washed 3× in distilled water and incubated in 2% CoCl_2_ for 5 min. After washing 3×, a 5% solution of ammonium sulphide was applied for 30 s before thorough washing in distilled water. Type II fibres stained dark, while type I fibres were light. Muscle sections were imaged on a Nikon Eclipse 50i microscope at 10×, with serial images encompassing the whole muscle section captured. Individual muscle fibres were counted using ImageJ (v1.52) and the cell counter plug-in. A minimum of 250 fibres were counted per muscle section.

### Magnetic Resonance Imaging and Muscle Volume Analysis

Participants were placed feet first, supine into the MRI and instructed to relax during imaging to minimise motion artefacts. Participants remained supine for at least 10 min before scanning. A 1.5T MRI system (Avanto, Siemens Munich, Germany) was used to collect images of the whole leg, with a localiser scan used to align subsequent images to capture the whole of both legs. Since this cannot be acquired in a single slice pack, two sets of axial images were collected which are automatically composited by the system software. An imaging matrix of 512 × 235 with a resolution of 835 × 835 μm was acquired with a slice thickness of 5 mm using a turbo spin echo sequence with an echo time set to the minimum value of 12 ms and a repetition time of 568 ms to optimise the trade-off between imaging time and contrast for a proton density weighted image. A Siemens peripheral angiography coil was used to maximise the signal to noise ratio of the resulting images. MRI scans were analysed using Slicer (v4.10), with TA and MG individually segmented by pixel count method every third slice (representative images Figure 4A) before semi-automatically filling between slices to generate 3D muscle volumes for quantification (Figure 4B).

### Ultrasound and DXA Derived Muscle Mass

Ultrasound images were obtained while the participant was in a supine position for at least 10 min, with their leg extended and ankle relaxed (∼90°). This position was chosen to allow the TA and MG to be relaxed and imitates the knee and ankle position during the MRI scan. TA and MG were scanned at 30% of their length on the mid-sagittal line. TA was measured from the mid-point of the knee on the anterior side of the leg to the fibula end (ankle) and MG from the inner knee crease to the ankle. These anatomical landmarks were chosen to standardise scanning locations and take into account variation in leg length between participants. Images were captured using B-mode ultrasonography (Mylab 70, Esaote Biomedica, Italy), with the transducer aligned in the fascicle plane (Figures 5A,B). Quantification of muscle thickness, fascicle length and pennation angle in each muscle was performed using ImageJ (Version 1.53), with muscle thickness determined through measurement of the distance between the superficial and deep aponeuroses. An average measurement between three images per muscle was used for quantification.

DXA scans (GE Medical Systems-Lunar Prodigy, United States) were undertaken to determine fat free mass (FFM) and lean mass. Participants wore loose comfortable clothing with no metal or plastic components. The system was calibrated before use with a QA phantom block, with spine phantoms run bi-monthly to assess reproducibility and accuracy of the system. Regional analysis was achieved manually, with the lower leg segmented from below the patella, encompassing the remaining portion of the leg.

### Statistical Analysis

All analyses were performed on GraphPad Prism (v8.4.3), with columns depicting the mean and individual values shown. Box and whisker plots are min to max values (whiskers) with the mean depicted as “+” and box margins the 25th and 75th percentiles. Repeated measures two-way ANOVA with multiple comparisons (time vs. condition) were conducted to determine differences in pre and post immobilisation and Tukey *post hoc* analysis to compare between limbs. Delta changes in volume was assessed by paired *t*-test. Correlative analysis was undertaken by linear regression analysis. Results are discussed as mean ± SEM. Statistical significance was accepted at an alpha level of less than 0.05.

## Results

### Fibre Type Distribution and Body Composition

**FIGURE 2:**
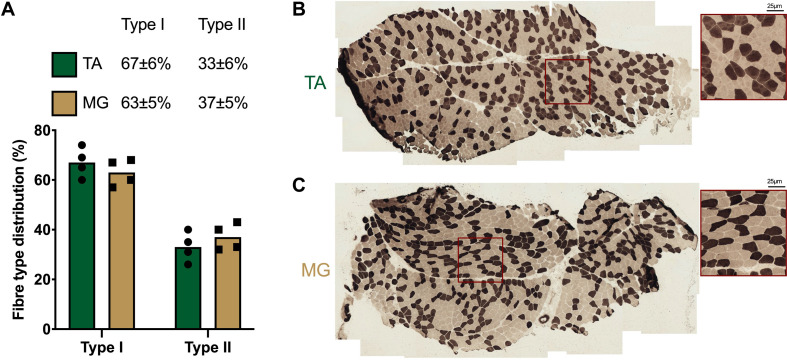
**(A)** Quantification and representative images of muscle fibre type distribution in **(B)** *tibialis anterior* (TA) and **(C)** *medial gastrocnemius* (MG). Type I fibres: light; Type II fibres: dark. Values are mean, with individual values.

**FIGURE 3:**
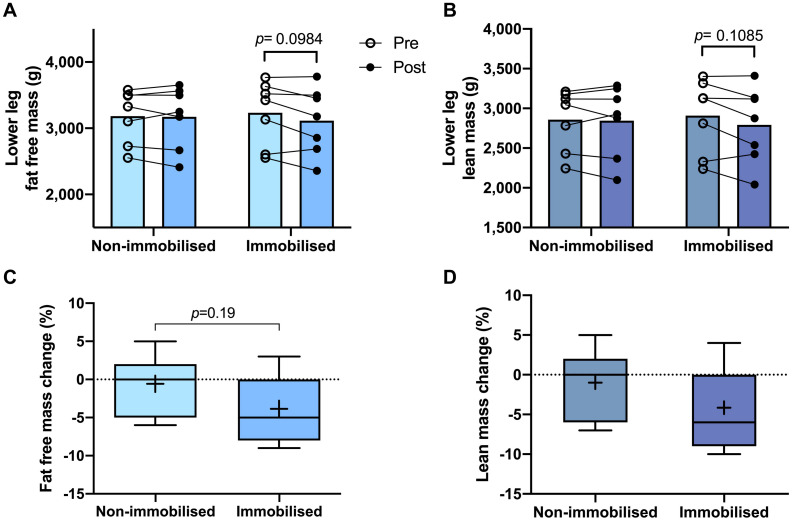
Pre and post 15 day unilateral leg immobilisation lower leg fat free mass **(A)** and lean mass **(B)** in non-immobilised and immobilised legs. Corresponding percentage changes in fat free mass **(C)** and lean mass **(D)**. Mean values are denoted as +, with box margins the 25th and 75th percentile and whiskers min to max values. Pre and post analysis measured by 2-way ANOVA, percentage change by paired *t*-test.

Both non-immobilised TA and MG were predominantly comprised of type I fibres (TA 67 ± 3%, MG 63% ± 3; Figures 2A–C). Prior to ULI, there were no differences between legs for either lower leg FFM (non-immobilised 3,184 ± 153 g vs. immobilised 3,234 ± 185 g) or lean mass (non-immobilised 2859 ± 146 g vs. immobilised 2,908 ± 176 g), with the non-immobilised leg remaining unchanged (*p* = 0.97) post ULI intervention (Figures 3A,B). In the immobilised leg a small non-significant decrease was observed in lower leg FFM (Post ULI: 3,115 ± 191 g, *p* = 0.098, −3.9%) and lean mass (Post ULI: 2.791 ± 181 g, *p* = 0.11, −4.1%) (Figures 3C,D). Lower leg bone mineral content remained unchanged following ULI (data not shown).

### MRI Derived Muscle Volume

**FIGURE 4:**
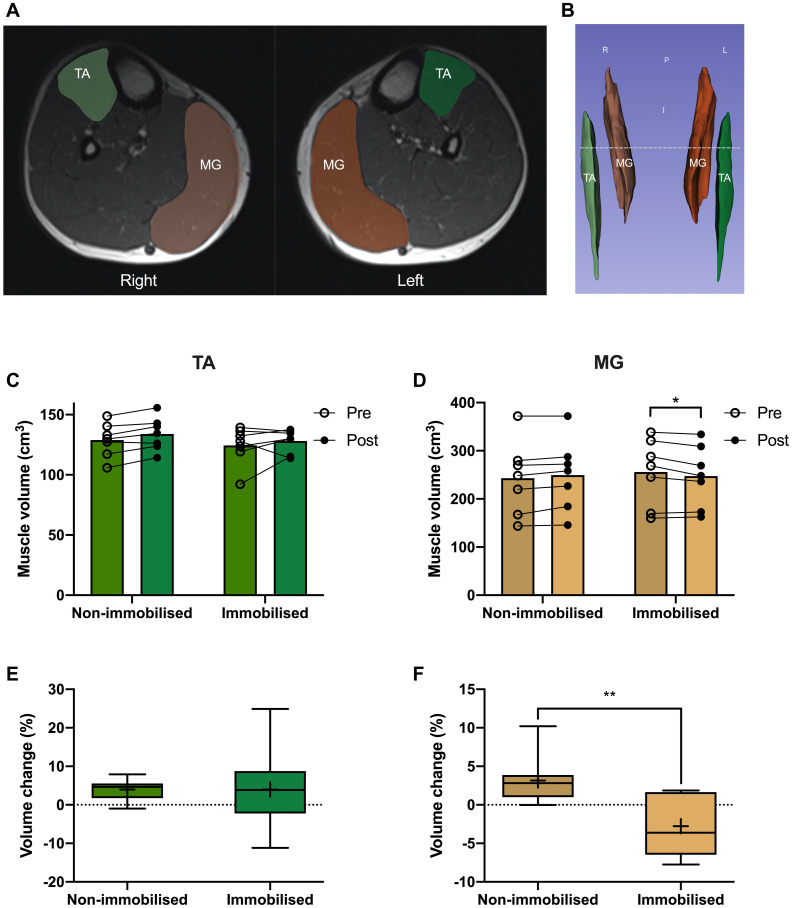
**(A)** MRI image of *tibialis anterior* (TA; green) and *medial gastrocnemius* (MG; orange), and representative 3D segmentation volume analysis **(B)**, dotted line denotes MRI slice. Pre and post 15 day unilateral leg immobilisation TA **(C)** and MG **(D)** volume in non-immobilised and immobilised legs. Muscle volume changes compared to baseline values in TA **(E)** and MG **(F)**. Mean values are denoted as +, with box margins the 25th and 75th percentile and whiskers min to max values. *p < 0.05, **p < 0.01 between indicated groups. Pre and post analysis measured by 2-way ANOVA, volume change by paired *t*-test.

There were no differences in TA or MG volumes between non-immobilised and immobilised legs prior to ULI. Furthermore, TA remained unchanged in response to ULI in both the non-immobilised (Pre 129 ± 5.4 cm^3^ vs. post 134 ± 5.2 cm^3^) and immobilised (Pre 124.5 ± 6 cm^3^ vs. post 128.2 ± 3.7 cm^3^) legs (Figures 4C,D). MG volume in the non-immobilised leg also remained unchanged (Pre 243.1 ± 28.8 cm^3^ vs. post 249.6 ± 27.9 cm^3^), although there was a significant decrease in the immobilised leg (Pre 255.9 ± 26.3 cm^3^ vs. post 247.5 ± 24.2 cm^3^, *p* = 0.01). While percentage volume change from baseline remained stable in TA (non-immobilised 3.9 ± 1.1% vs. immobilised 3.9 ± 4.3%), there was a significant decrease in MG with ULI (non-immobilised 3.2 ± 1.3% vs. immobilised −2.8 ± 1.4%, *p* = 0.009) (Figures 4E,F).

### Ultrasound Measures

**FIGURE 5:**
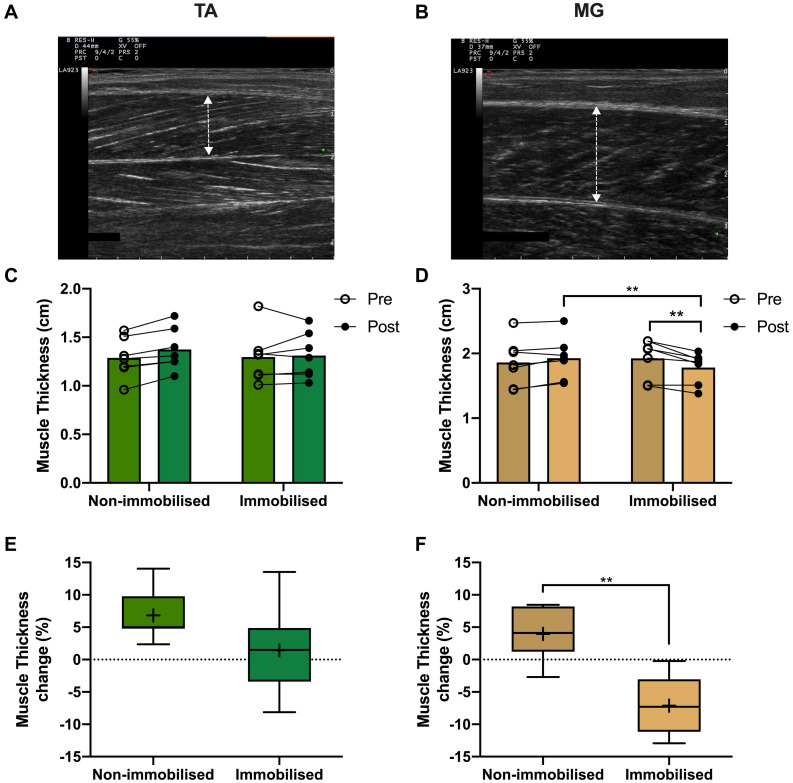
Representative muscle thickness ultrasound image of **(A)** tibialis anterior (TA) and **(B)** medial gastrocnemius (MG). Pre and post 15-day unilateral leg immobilisation TA **(C)** and MG **(D)** muscle thickness in non-immobilised and immobilised legs. Muscle thickness changes compared to baseline values in in TA **(E)** and MG **(F)**. Mean values are denoted as +, with box margins the 25th and 75th percentile and whiskers min to max values. **p < 0.01 between indicated groups. Pre and post analysis measured by 2-way ANOVA, percentage change by paired *t* test.

There were no differences in muscle thickness between non-immobilised or immobilised TA prior to ULI (Figure 5C and Table 1). In response to ULI, TA muscle thickness remained unchanged in both legs, while muscle thickness significantly decreased in immobilised MG (Pre 1.93 ± 0.12 cm vs. post 1.78 ± 0.09 g, *p* = 0.007), resulting in a significantly reduced thickness compared to the non-immobilised leg (*p* = 0.007, Figure 5D and Table 1). While percentage changes in TA muscle thickness remained unchanged with ULI, MG muscle thickness was significantly decreased between legs (non-immobilised −2.7 ± 1.5% vs. immobilised −12.9 ± 1.6%, *p* = 0.002) (Figures 5E,F). In TA, fascicle length and pennation angle were comparable between both legs pre and post immobilisation. While pennation angle was significantly reduced between legs in MG muscles post immobilisation (non-immobilised 35.41 ± 2.22 vs. immobilised 29.27 ± 2.11, *p* = 0.05, Table 1), no significant difference was observed within the immobilised leg.

**TABLE 1:** Muscle ultrasound measures.

*tibialis anterior* (TA)	Non-immobilised		Immobilised	
	Pre	Post	Pre	Post
Muscle thickness (cm)	1.29 ± 0.08	1.37 ± 0.08	1.3 ± 0.1	1.31 ± 0.09
Fascicle length (cm)	6.85 ± 0.4	7.33 ± 0.32	6.41 ± 0.56	7.05 ± 0.08
Pennation angle (°)	9.83 ± 0.58	10.04 ± 0.94	9.69 ± 0.97	8.66 ± 0.87

***medial gastrocnemius* (MG)**	**Non-immobilised**		**Immobilised**	
	**Pre**	**Post**	**Pre**	**Post**

Muscle thickness (cm)	1.86 ± 0.14	1.93 ± 0.12	1.92 ± 0.11	1.78 ± 0.09**^††^
Fascicle length (cm)	3.38 ± 0.21	3.69 ± 0.4	3.64 ± 0.18	3.94 ± 0.41
Pennation angle (°)	33.54 ± 1.13	35.41 ± 2.22	33.16 ± 1.89	29.27 ± 2.11^†^

Values are mean ± SEM. **Significantly different from pre immobilisation within that leg *p* < 0.01, ^†^p < 0.05, ^†^^†^*p* < 0.01 significantly different between legs at that time-point.

### Correlative Analysis

**FIGURE 6:**
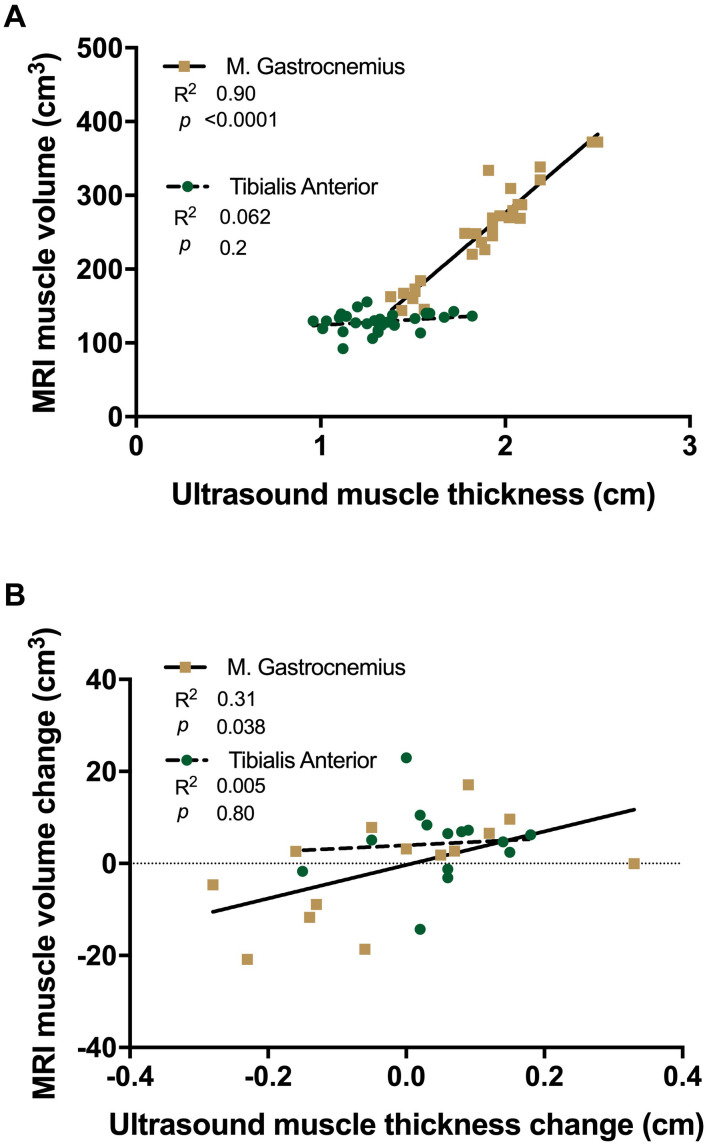
Linear regression analysis of ultrasound derived muscle thickness vs. MRI muscle volume **(A)** and changes with 15 day unilateral leg immobilisation **(B)** in *tibialis anterior* (TA, green) and *medial gastrocnemius* (MG, gold).

A significant positive correlation was shown between MRI based muscle volume and U/S derived muscle thickness measures in MG (R^2^ = 0.90, *p* = 0.0001, Figure 6A), which was not significant in TA (R^2^ = 0.062, *p* = 0.2). There was a significant correlation in the absolute amount of muscle loss between MRI and U/S measures, with atrophying MG demonstrating a positive correlation (R^2^ = 0.31, *p* = 0.038, Figure 6B), whereas there was no association in the atrophy resistant TA (R^2^ = 0.005, *p* = 0.8).

## Discussion

While crucial insights into the loss of skeletal muscle mass during DA have been gained with the use of varying disuse models (i.e., bed rest, ULI, casting), the core mechanisms of DA remain incompletely defined (Rudrappa et al., 2016; Kilroe et al., 2020). We posited that divergent atrophy responses between individual muscles, currently not understood, may provide clarity on the regulatory mechanisms of DA (Belavý et al., 2009), in relation to core processes driving DA against those reflecting an altered metabolic state, i.e., processes not strongly linked to DA *per se*. ULI remains one of the more feasible models to study the repercussions and mechanisms of DA in both young and old individuals (de Boer et al., 2007; Dideriksen et al., 2016). Here, we show it achievable and effective to simultaneously investigate atrophy susceptible (MG) and resistant (TA) muscles in response to a ULI paradigm in humans. Furthermore, we demonstrate the utility of this method, employing multiple standard imaging techniques (DXA, U/S, MRI) to examine changes in muscle mass.

Although ULI-induced decreases in lower-leg FFM, and lean mass were shown by DXA, the change was relatively small (both ∼−4%). In addition, these decreases reflect the measurement of gross changes in the lower-leg (rather than of individual muscles), encompassing multiple muscle groups (including TA and MG) with varying degrees of atrophy susceptibility (Miokovic et al., 2012). Moreover, direct comparison between DXA and MRI measurements have demonstrated a tendency of DXA to over-estimate lean mass (Maden-Wilkinson et al., 2013), negatively biasing measurements of smaller masses (i.e., lower limb). However, the convenience and utility of DXA still provides valuable insight into gross physiological changes occurring in the limb (Visser et al., 1999), with previous investigations reporting decreases in DXA derived whole leg lean mass following bed rest or ULI (Wall et al., 2014; English et al., 2016). The present study was not powered to detect change in DXA outcomes, which might have resulted in underpowered analysis of smaller tissue mass decreases in the lower limb, as compared to that of larger masses (e.g., the thigh). For future trials using aRaS, *post-hoc* analysis determined that significance in FFM changes of the lower limb would be shown with a minimum of 14 participants (α error probability = 0.05, power (1-β) = 0.8, effect size = −0.841), a common range for physiological studies (de Boer et al., 2007; Wall et al., 2014; Kilroe et al., 2020). Thus, utilisation of DXA derived measurements in a sufficiently powered study will provide valuable assessment of lower limb tissue mass changes following ULI.

The measurement of specific muscles rather than limb segments allows individual characterisation of atrophy responses. Proof-of-concept that the current ULI paradigm permits simultaneous investigation of distinct muscles requires the ability to substantiate and distinguish differing disuse responses. As hypothesised, we confirmed MG volume and thickness decreases following 15 days ULI, in keeping with previous studies of bed rest (Belavý et al., 2009), whereby MG exhibited a loss in muscle thickness and pennation angle (Abe et al., 1997; de Boer et al., 2008). Accurate assessment of such changes is physiologically important as reductions in physiological CSA and muscle volume relate to declining functional outcomes. Moreover, previous investigations identified similar degrees of quadriceps muscle loss following a period of ULI (Dirks et al., 2014; Kilroe et al., 2020). We also exposed a TA atrophy resistance phenomenon, with no reciprocal decreases in either muscle volume or thickness, similar to previous bed-rest observations (de Boer et al., 2008; Belavý et al., 2009; Miokovic et al., 2012). To our knowledge, this is the first study to utilise ULI to investigate DA responses of lower limb muscles, confirming our empirical observations of relative atrophy susceptibility between MG and TA muscles.

Use of U/S to conveniently and accurately quantify muscle mass (Reeves et al., 2004) has become a ubiquitous tool to investigate muscle adaptation in health and disease (de Boer et al., 2008; Nijholt et al., 2017; Franchi et al., 2018). In our experimental paradigm, correlation between MG thickness and MRI-derived muscle volume (regarded as the gold-standard) robustly demonstrates the ability to accurately determine muscle size by U/S, similar to that in previous studies (Smith et al., 2017; Franchi et al., 2018). However, while TA and MG thickness and CSA have previously been linked (Smith et al., 2017), it is important to note the absence of correlation between TA volume and thickness in this study. This is likely due to the longer geometric shape and a smaller more consistent CSA of TA compared to MG (Fukunaga et al., 1992). Additionally, the lack of muscle loss correlation in TA is not surprising as no atrophy was detectable. Ultrasound measures for each muscle taken at 30% of the whole length is able to reliably assess changes occurring in the muscle belly (i.e., thickness, fascicle length and pennation angle), where changes may be sufficiently detected.

Investigation into the mechanisms underpinning DA has identified systematic suppression of muscle protein synthesis (MPS) as the predominant factor responsible for atrophy during prolonged disuse (Gibson et al., 1987; Ferrando et al., 1996). Further, this disruption in MPS occurs rapidly (i.e., in 5–14 days) with a coordinated suppression of both post-absorptive and post-prandial MPS rates occurring (Wall et al., 2013, 2016). Interestingly, unstimulated rates of MPS are comparable between muscles (e.g., the soleus and VL) despite differing fibre type composition, yet they display varied responses to stimuli (i.e., resistance exercise) (Trappe T. A. et al., 2004). While MPS in MG muscle is likely to be suppressed in response to disuse, evidenced by reductions in muscle thickness and number of parallel sarcomeres (de Boer et al., 2008), perturbations in protein turnover in different muscles (specifically in this instance MG and TA) are currently unclear. Furthermore, temporal characterisation of MPS responses to disuse may explain the initially rapid losses in muscle mass followed by a slower rate observed in some muscles (Belavý et al., 2009; Kilroe et al., 2020). Here, we have developed and demonstrated the applicability of two distinct muscle biopsy techniques (i.e., conchotome and microneedle) in both TA and MG muscles, that may be further integrated with stable isotope tracer techniques to probe MPS protein sub-fraction or OMIC responses to disuse (Wilkinson et al., 2015; Miller et al., 2019). Thus, inclusion of muscle biopsies in conjunction with the aRaS paradigm should permit comparative analysis of MPS, MPB (e.g., using pulse-chase tracer approaches; Zhang et al., 2002) and also the underpinning molecular responses. In sum, we posit metabolic perturbations in atrophy resistant muscle(s) will present a more informative control than a non-immobilised muscle, to tease out the core mechanisms of DA.

In terms of more fundamental muscle physiology, atrophy susceptibility has previously been attributed to the fibre type composition of a given muscle. Our current analyses confirm the previously limited human TA and MG fibre composition data, as being predominantly type I (Henriksson-Larsen et al., 1983). Importantly, general fibre type composition between TA and MG was comparable, demonstrating fibre type alone is highly unlikely to explain gross differential atrophy susceptibility between these muscles. Indeed, variance in type II fibre composition (i.e., IIa/IIb) is also unlikely to resolve atrophy susceptibility due to their lesser frequency (i.e., ∼35%), and since disuse induced fibre atrophy is greater in type I than type II fibres in soleus and MG muscles (Fitts et al., 2010). Similarly, reductions in in VL CSA following bed rest were more pronounced in type I fibres, with a shift in composition toward faster fibres (i.e., type IIx) (Brocca et al., 2012). Nonetheless, further investigations are warranted to elucidate CSA fibre type specific responses in DA, with aspects of fibre type shifting and capillarisation of particular importance. Additionally, aspects of muscle fibre innervation or denervation may provide an important facet of divergent muscle atrophy responses. Potential fibre losses and successful remodelling of motor units may provide important insight into DA susceptibility of muscle, in line with observations in aged muscle (Piasecki et al., 2018). On a further technical note, it is likely TA sparing may not be due slight plantar flexion and the resulting stretch-induced stimulation observed in previous investigations during supine bed rest (de Boer et al., 2008). However, in the aRaS model, with the ankle immobilised in a neutral position, alongside a fixed knee brace (75° flexion) and air-boot allowed ease of locomotion with no weight-bearing, while ensuring not TA or MG lengthening could occur. Finally, a potential limitation that must be noted is potential blood pooling in the immobilised leg, while participants remained supine for a minimum of 10 min before MRI and U/S scans to aid in fluid re-equilibration, longer periods may be required.

## Conclusion

In conclusion, aRaS provides a convenient and effective model to study DA, while permitting analysis of divergent mechanisms driving distinct muscle atrophy through direct comparison between MG and TA muscles with the addition of muscle biopsies to probe the molecular and physiological regulation of DA. If similar mechanistic responses are present in both atrophied and non-atrophied muscles it may indicate these are of lesser consequence in the progression of muscle loss; in contrast diverging mechanisms are more likely to represent core DA processes. Ultimately, greater understanding of divergent atrophy responses may lead to development of novel therapeutics to maintain muscle mass.

## Data Availability Statement

The raw data supporting the conclusions of this article will be made available by the authors, without undue reservation.

## Ethics Statement

The studies involving human participants were reviewed and approved by the University of Nottingham Faculty of Medicine and Health Sciences Research Ethics Committee. The patients/participants provided their written informed consent to participate in this study.

## Author Contributions

BP, PA, DW, KS, MP, and CS conceived and designed the study. JB, EH, and TI organised and carried out the clinical experiments and analysis. RM, AS, and CS performed MRI scanning. JB, BP, and PA wrote the manuscript. All authors gave their approval of the version of the article to be published.

## Conflict of Interest

The authors declare that the research was conducted in the absence of any commercial or financial relationships that could be construed as a potential conflict of interest.
